# Remote auditory assessment using Portable Automated Rapid Testing (PART) and participant-owned devicesa)

**DOI:** 10.1121/10.0013221

**Published:** 2022-08-01

**Authors:** E. Sebastian Lelo de Larrea-Mancera, Trevor Stavropoulos, Audrey Anna Carrillo, Sierra Cheung, Yue J. He, David A. Eddins, Michelle R. Molis, Frederick J. Gallun, Aaron R. Seitz

**Affiliations:** 1 Psychology Department, University of California, Riverside, 900 University Avenue, Riverside, California 92507, USA; 2 Brain Game Center, University of California, Riverside, 1201 University Avenue #204, Riverside, California 92507, USA; 3 University of South Florida, 4202 East Fowler Avenue, Tampa, Florida 33620, USA; 4 Oregon Health and Science University, 3181 Southwest Sam Jackson Park Road, Portland, Oregon 97239-3098, USA

## Abstract

Remote testing of auditory function can be transformative to both basic research and hearing healthcare; however, historically, many obstacles have limited remote collection of reliable and valid auditory psychometric data. Here, we report performance on a battery of auditory processing tests using a remotely administered system, Portable Automatic Rapid Testing. We compare a previously reported dataset collected in a laboratory setting with the same measures using uncalibrated, participant-owned devices in remote settings (experiment 1, *n* = 40) remote with and without calibrated hardware (experiment 2, *n* = 36) and laboratory with and without calibrated hardware (experiment 3, *n *=* *58). Results were well-matched across datasets and had similar reliability, but overall performance was slightly worse than published norms. Analyses of potential nuisance factors such as environmental noise, distraction, or lack of calibration failed to provide reliable evidence that these factors contributed to the observed variance in performance. These data indicate feasibility of remote testing of suprathreshold auditory processing using participants' own devices. Although the current investigation was limited to young participants without hearing difficulties, its outcomes demonstrate the potential for large-scale, remote hearing testing of more hearing-diverse populations both to advance basic science and to establish the clinical viability of auditory remote testing.

## I. INTRODUCTION

Valid and reliable remote evaluation of hearing ability is increasingly becoming an essential need for researchers and clinicians (Peng e*t al.*, 2022; Bokolo, 2021; Kim *et al.*, 2021). These issues have been brought to the forefront during social distancing associated with the COVID-19 pandemic; nevertheless, the advantages of remote testing are timeless and manifold. Reliable psychoacoustical testing outside of the confines of specialized audiometric laboratories could engender transformative changes in data collection that would increase accessibility and inclusion for both basic research and clinical auditory assessment (Woods *et al.*, 2015). This transformation is within reach as new technological developments afford high-quality audio generation software that can be implemented on platforms ranging from mobile phones to tablets and personal computers (see Gallun, 2020).

While initial successes collecting equivalent data in the lab vs remotely (e.g., Whitton *et al.*, 2016; Merchant *et al.*, 2021; Mok *et al.*, 2021) suggest that at least certain tests can be reliably conducted remotely, many obstacles remain to robust and reliable remote data collection that maintains data quality suitable for clinical or research purposes. Issues of calibration, accurate stimulus generation and playback, and interference by the external environment during testing remain significant concerns (Woods *et al.*, 2017; Peng *et al.*, 2022; Kim *et al.*, 2021; Kopun *et al.*, 2022). Still, the implementation of remote testing of auditory abilities, with a range of assessments and parametric specifications, is proliferating (e.g., Shafiro *et al.*, 2020; Mok *et al.*, 2021; Hisagi *et al.*, 2020). This rapid growth in the alternatives for remote auditory testing creates an environment where it may be challenging to compare findings across different systems, as has been the case for the assessment of working memory (Pergher *et al.*, 2020). To address these issues, we developed an app-based platform called Portable Automatic Rapid Testing (PART) that is freely available for a wide range of mobile and desktop/laptop operating systems. PART can be used to design and run a large set of psychophysical tests with laboratory-grade quality (Gallun *et al.*, 2018), allowing experimental consistency to be maintained across a wide range of tasks. PART's accessibility and versatility point toward its substantial potential to address the growing need for valid and reliable psychophysical testing of diverse auditory abilities.

Larrea-Mancera *et al.* (2020) reported data from a diverse set of auditory measures with potential clinical use that were collected in a laboratory setting using PART. The measures collected included spatial, spectral, and temporal sensitivity and signal detection in competition of both synthetic stimuli (tones and noises) and speech [see also Srinivasan *et al.* (2020), Diedesch *et al.* (2021), and Gallun *et al.* (2022)]. These assessments of auditory function are thought to be related to different aspects of hearing in real-world conditions. For example, some studies have found that sensitivity to spectro-temporal modulation (STM) explains an additional 40% of the variance in ability to understand speech beyond that provided by the pure tone audiogram alone (Bernstein *et al.*, 2013; Mehraei *et al.*, 2014). Additionally, frequency modulation (FM) detection and gap detection tasks have been associated with ability to detect temporal fine structure (TFS) cues—also shown to be important for speech understanding (Gallun *et al.*, 2014; Hoover *et al.*, 2019). However, there is disagreement across studies as to how much variance these factors explain [see, for example, Füllgrabe *et al.* (2015), Bernstein *et al.* (2016), and Gallun *et al.* (2022)]. For this reason, it is important to collect more data from larger data sets, using easily comparable methods. PART is designed specifically to support such replicable, large-scale, multisite studies.

There are a number of potential difficulties with remote testing, as described in detail in the report of the Acoustical Society of America's Taskforce on Remote Testing (Peng *et al.*, 2022). Most importantly, the equipment and the testing environment may differ significantly from that used in the laboratory. To address the issue of the equipment, we have tested PART in the laboratory using the same type of equipment that would be used for remote testing (Larrea-Mancera *et al.*, 2020) and found good correspondence with data published from experiments using standard laboratory equipment. This shows that consumer-grade equipment can be used to test a wide range of suprathreshold abilities. In this study, we take the next step, to examining changes in the listening environment and using a wider range of consumer devices. Potential differences between the calibrated systems used previously and participant-owned devices included differences in the output levels of the devices, as well as variations in the frequency response of the transducers and the degree to which the level and phases of the headphones are matched across the ears. The difference in output levels was addressed in one of the experiments reported in Larrea-Mancera *et al.* (2020). In that experiment, two different headphones were used, but the calibration settings of the devices were not changed between testing with the two headphone types. This resulted in a 14-dB decrease in output level for the uncalibrated headphone, but there was no significant difference in performance between the two transducer types. This supports the extension here to participant-owned equipment, but an important question to be addressed is whether or not between-ear differences might be more likely in this data set and whether or not this would result in reduced performance on the binaural tests.

Here, we report data from a series of experiments that examine the validity and reliability of remote assessment via PART using the battery of auditory processing tests from Larrea-Mancera *et al.* (2020) [see also Srinivasan *et al.* (2020), Diedesch *et al.* (2021), and Gallun *et al.* (2022)]. We compare the results of Larrea-Mancera *et al.* (2020)—collected in laboratory settings—to remote assessment using uncalibrated, participant-owned devices (experiment 1, *n* = 40), remote assessment with and without standardized calibrated hardware (experiment 2, *n* = 36), and laboratory assessment with and without calibrated hardware (experiment 3, *n* = 58). Data obtained were largely consistent across datasets, showing the validity of remote testing while maintaining similar reliability. Overall, these data support the use of remote testing of auditory abilities evaluated by psychophysical tests implemented on widespread technologies such as tablet computers and smart phones.

## II. METHODS

The methods of Larrea-Mancera *et al.* (2020) were duplicated as closely as possible; modifications were limited primarily to those necessary for remote data collection. Tests were exact replications of the previous study in terms of parameters, instructions, and order of presentation (with the exception of the notch-noise tasks that were excluded due to a technical error in parameterization). Exact stimulus levels presented to participants [dB sound pressure level (SPL)] are not available for data collected with uncalibrated devices; however, given that PART specifies output intensity in dB units, we refer to the specified levels in dB to facilitate comparisons across datasets. As such, presentation levels given for the uncalibrated systems should be understood to refer to the nominal output level specified in PART rather than the actual output level in dB SPL.

Unlike in the normative study of Larrea-Mancera *et al.* (2020), here, we included an audibility test to exclude participants who were unable to achieve minimum audibility, either due to poor equipment or individual factors such as hearing loss or lack of attentional focus. Participant elimination from analysis was based on a failure to achieve a minimum level of performance in two of three tests (a 40-dB threshold for 2-kHz or broadband noise detection or a 50-dB threshold for correct performance on sentences from the coordinate response measure (CRM) (Bolia *et al.*, 2000) corpus produced by a single talker). The minimum audibility cutoffs were selected to be just below the levels used in the signal in competition tests (45 dB for pure tones and 65 dB for speech). Elimination criteria were applied after participants had completed all testing. Moreover, outlying datapoints beyond 3 standard deviations (SDs) from the normative dataset mean (Larrea-Mancera *et al.*, 2020) were excluded from the main analysis, as this was also considered evidence of equipment failures or transient individual factors such as lack of attention to the testing. Additional analyses including the outlying datapoints (but not eliminated subjects) are included in the supplemental materials.^1^

### A. Participants

Participants were 134 undergraduate students from the University of California, Riverside, who either received course credit or were paid $10/h. for their participation. Experiment 1 consisted of 40 listeners [24 female, mean (*M*) age = 20.1 years, SD =* *2.2 years]; experiment 2 consisted of 36 listeners (23 female, *M* age = 21.4 years, SD = 3 years); and experiment 3 consisted of 58 listeners (31 female, *M* age = 20.1 years, SD = 2.9 years). Four participants failed to achieve minimum performance on the audibility sub-battery and were excluded from experiment 1; none of the participants in experiments 2 and 3 were excluded based on audibility. All participants reported normal hearing and vision and no history of psychiatric or neurological disorders, and all provided electronic informed consent as approved by the University of California, Riverside, Human Research Review Board.

### B. Materials

Participants completed experiment 1 remotely, and all procedures were conducted using their own uncalibrated smartphones or tablets [34 Apple (Cupertino, CA) iOS and 6 Android devices] and with their own headphones or earbuds (categorized for descriptive purposes as either Apple wired, Apple wireless, or other). Participants using Android devices employed headphones with a variety of wired, wireless, and hybrid features (*N* = 5), one made by Apple (wired). Participants using iOS devices (*N* = 34) employed Apple wired (*n* = 8), Apple wireless (*n* = 16), or other headphones (*N* = 10). Participants were encouraged to complete the experimental sessions at home and were instructed to make sure they were in a quiet location where they would not be disturbed. To streamline user experience, a variant of PART called BGC Science was employed. BGC Science uses the same codebase as PART but allows participants to enter a server code that auto-configures the appropriate test battery. Data were encrypted and securely transmitted to an Amazon Web Services server (Amazon Web Services, Seattle, WA). No participant identifiers were transmitted or stored with the data.

Experiment 2 was also conducted remotely, but a calibrated equipment set identical to that used in Larrea-Mancera *et al.* (2020)—an iPad (sixth generation; Apple) and a pair of Sennheiser (Wedemark, Germany) 280 Pro headphones—was delivered to participants' homes. Participants were randomly assigned to use the calibrated equipment in either the first or the second session and their own uncalibrated equipment in the other (28 Apple iOS and 8 Android devices). Participants using Android devices (*N* = 8) employed Apple wired headphones (*n* = 1) or other brand headphones (*n* = 7). Participants using Apple devices (*N* = 28) employed Apple wired headphones (*n* = 10), Apple wireless headphones (*n* = 12), or other brand headphones (*n* = 6). In experiment 3, the order of test sessions using calibrated and uncalibrated (47 Apple iOS and 11 Android devices) systems was randomized, and all test procedures were conducted in the laboratory. Participants using Android devices (*N* = 11) employed Apple wired headphones (*n* = 1) or other brand headphones (*n* = 10). Participants using Apple devices (*N* = 47) employed Apple wired headphones (*n* = 10), Apple wireless headphones (*n* = 26), or other brand headphones (*n* = 11).

### C. Assessments

All three experiments used the same task structures as in Larrea-Mancera *et al.* (2020). For the speech in competition tasks (described below) that use the CRM corpus (Bolia *et al.*, 2000), data were collected using a colored number grid (five colors and numbers 0–9). For all other tasks, data were collected using a four-interval, two-cue, two-alternative forced-choice (2-Cue 2-AFC) paradigm. In each trial, four squares were presented on a touchscreen and were lit sequentially from left to right to coincide with the presentation of four sounds. Target sounds were presented randomly to coincide with either the second or third interval; a standard stimulus was presented in each of the remaining intervals—the first, the last, and whichever of the middle two did not contain the target. Participants indicated their responses by tapping the square corresponding to the target interval, either the second or third. Because the standard stimulus was always presented in the first and last intervals, the target stimulus was always preceded and followed by a standard stimulus.

#### 1. Minimum audibility

##### a. Pure tone detection in quiet.

A progressive tracking algorithm was used in which a 100-ms 2-kHz tone was initially presented at a level of 70 dB and was then reduced every three trials in 5-dB steps until either a presentation level of 5 dB was reached or three errors occurred over six trials.

##### b. Broadband noise detection in quiet.

A progressive track was used to reduce the level of a 100-ms white noise stimulus initially presented at 70 dB and reduced by 5 dB every three trials. The same stopping rule was applied as for pure tone detection in quiet: a 5-dB presentation level or three errors over six trials.

##### c. Single talker speech identification.

A single talker was chosen randomly from the first three male talkers in the CRM corpus (Bolia *et al.*, 2000), and one randomly selected sentence spoken by that talker was presented on each trial. The sentences all included the call sign “Charlie” and two keywords: a number and a color. Participants indicated their responses by selecting a single button on a graphical display composed of a grid of colored numbers. Performance was measured based on correct identification of both color and number. The same initial presentation level and stopping rule were used as for pure tone detection and broadband noise detection tasks.

#### 2. TFS

##### a. Temporal gap.

In this 2-Cue 2-AFC task, based on Hoover *et al.* (2019), each of the four intervals contained two 4-ms tone bursts (cropped Gaussians) presented diotically at a level of 80 dB. Tone bursts had a carrier frequency of 0.5 kHz. Target intervals contained a brief silent gap between the end of the first burst and the beginning of the second. The gap duration in the target interval was initially 20 ms and was varied according to a two-stage adaptive tracking algorithm that modified gap duration on an exponential scale by steps with a factor of 2^1/2^ in the first stage and by steps with a factor of 2^1/10^ in the second stage. The minimum gap duration was 0 ms, and the maximum gap duration was 100 ms.

##### b. Diotic FM.

This FM detection task, also based on the method of Hoover *et al.* (2019), was modified from the work of Grose and Mamo (2010). In all four intervals, tones with a carrier frequency between 460 and 550 Hz and a duration of 400 ms were presented diotically (identically at the two ears) at a level of 75 dB. Carrier frequencies were roved across intervals to reduce the informativeness of the presence of spectral energy outside of the standard carrier frequency range that can be caused by FM of sufficient depth. The target interval contained in-phase FM at a rate of 2 Hz and with a variable depth. Participants were instructed to select the interval containing the FM. A two-stage adaptive tracking algorithm was used to adjust the modulation depth on an exponential scale with descending first-stage step sizes of 2^1/2^ and second-stage step sizes of 2^1/10^ starting at 6 Hz with a minimum value of 0 Hz and a maximum value of 10 000 Hz.

##### c. Dichotic FM.

This FM detection task (Hoover *et al.*, 2019; Grose and Mamo, 2010) used the same stimuli and tracking methods as in the diotic FM task, but in this case, the FM was inverted in phase between the ears, so that as the frequency increased at one ear, it decreased at the other. This stimulus results in a time-varying interaural phase difference (IPD) that is perceived as a tone of fixed frequency varying in interaural location when the modulation rate is sufficiently small relative to the carrier frequency, such as the 2 Hz used here (Witton *et al.*, 2000). The same procedure and adaptive tracking were used as in the diotic FM task, as well as the same starting modulation range of 6 Hz.

#### 3. Spectro-temporal sensitivity

All three of these tasks employed a standard consisting of a 500-ms-long band of noise with a width of 0.4–8 kHz presented diotically at a level of 65 dB in each of four intervals. In each task described below, the 2-Cue 2-AFC procedure was used, and participants identified the target interval that contained temporal modulation (TM), spectral modulation (SM), or STM. The adaptive parameter (modulation depth) was applied on a logarithmic amplitude scale (dB) and was measured from the middle of the amplitude range to the peak amplitude as described in Stavropoulos *et al.* (2021). Modulation depth is expressed throughout the paper as M (dB). The adaptive tracking procedure used large descending steps of 0.5 dB and small descending steps of 0.1 dB, with a minimum modulation depth of 0.2 dB and a maximum modulation depth of 40 dB.

##### a. Temporal modulation.

The TM detection task, based on Viemeister (1979), required participants to detect sinusoidal temporal amplitude modulation (AM) at a rate of 4 Hz.

##### b. Spectral modulation.

The SM detection task, based on Hoover *et al.* (2018), required the detection of sinusoidal spectral modulation with random phase at a rate of 2 cycles per octave (c/o).

##### c. STM.

The STM detection task, based on Bernstein *et al.* (2013), required the detection of a stimulus that had both sinusoidal temporal AM at a rate of 4 Hz and sinusoidal spectral modulation with random phase at a rate of 2 c/o.

#### 4. Targets in competition

Sentences from the CRM corpus described in the audibility task (Bolia *et al.*, 2000) were used to set up two conditions of a three-talker speech-on-speech masking task. Sentences were spatialized according to condition using generic head-related transfer functions and the headphone-based presentation and scoring developed by Gallun *et al.* (2013). Target sentences all included the call sign “Charlie” and two keywords, a number and a color, fixed at a root mean square (rms) level of 65 dB. The target was presented simultaneously with two maskers, which were male talkers uttering sentences with different call signs, colors, and numbers in unison with each other and with the target.

##### a. SRM colocated.

All three sentences were presented from directly in front of the listener (colocated) in simulated space. Progressive tracking included 20 trials in which the maskers progressed in level from 55 to 73 dB in steps of 2 dB every two trials, with no stopping rule, as described in Gallun *et al.* (2013).

##### b. SRM separated.

The procedure, stimuli, and progressive tracking were identical to those in the colocated condition, with the exception that the maskers were presented from 45° to the left and right of the target talker in simulated space.

##### c. Spatial release from masking metric.

This was obtained by subtracting the masker level threshold obtained in the colocated condition from that obtained in the separated condition. Positive values indicate benefit from spatial cues in dB.

### D. Procedure of experiment 1

In this first experiment, each session began with a video call where participants completed a screening to ensure that sounds from the left and right channels were heard in the left and right ears, respectively. Participants were instructed to turn the volume to the maximum setting at the onset of testing and not to modify this until testing was completed. If participants did not comply, an automated message from PART notified participants if their volume was at the wrong setting and instructed them to adjust the volume to the correct setting. All participants then completed the audibility test. This was followed by two tests involving detection of tones in noise that are not described further due to the presence of programming errors. Following this, procedures were identical to those described in Larrea-Mancera *et al.* (2020), where participants conducted the TFS (three assessments), STM (three assessments), and the Targets in Competition sub-battery (two assessments) in pseudo-random order. Participants were encouraged to take short breaks between testing blocks. Each of the ten assessments took about 5 min to complete, resulting in test sessions of around 1 h. The second session was identical to the first and was always conducted on a different day no longer than a week after the first session.

## III. RESULTS OF EXPERIMENT 1

Results are divided into sections for the purpose of clarity. First, to evaluate thresholds for each assessment across two sessions, remote data are compared to the normative dataset from Larrea-Mancera *et al.* (2020) (Sec. III A). Then repeatability of measurements across sessions is evaluated by comparing differences across sessions between both studies (Sec. III B). Section III C then summarizes the findings of experiment 1 using standardized composite scores and discusses device and headphone effects. Of note, four participants were excluded due to failure to reach the minimum audibility cutoff in two of three tests. Audibility data are shown in the supplementary materials.^1^ Moreover, datapoints ±3 SD away from the normative dataset mean were rejected as outliers on the dichotic FM (5), diotic FM (4), TM (12), SM (7), STM (7), and SRM in the separated condition (1). Although these datapoints are excluded from analysis in the main report, all data including outliers are presented in the supplemental materials (see Fig. S1).^1^

### A. Are remotely administered thresholds with participant-owned devices comparable to those collected in the lab with calibrated equipment?

**FIG. 1.**
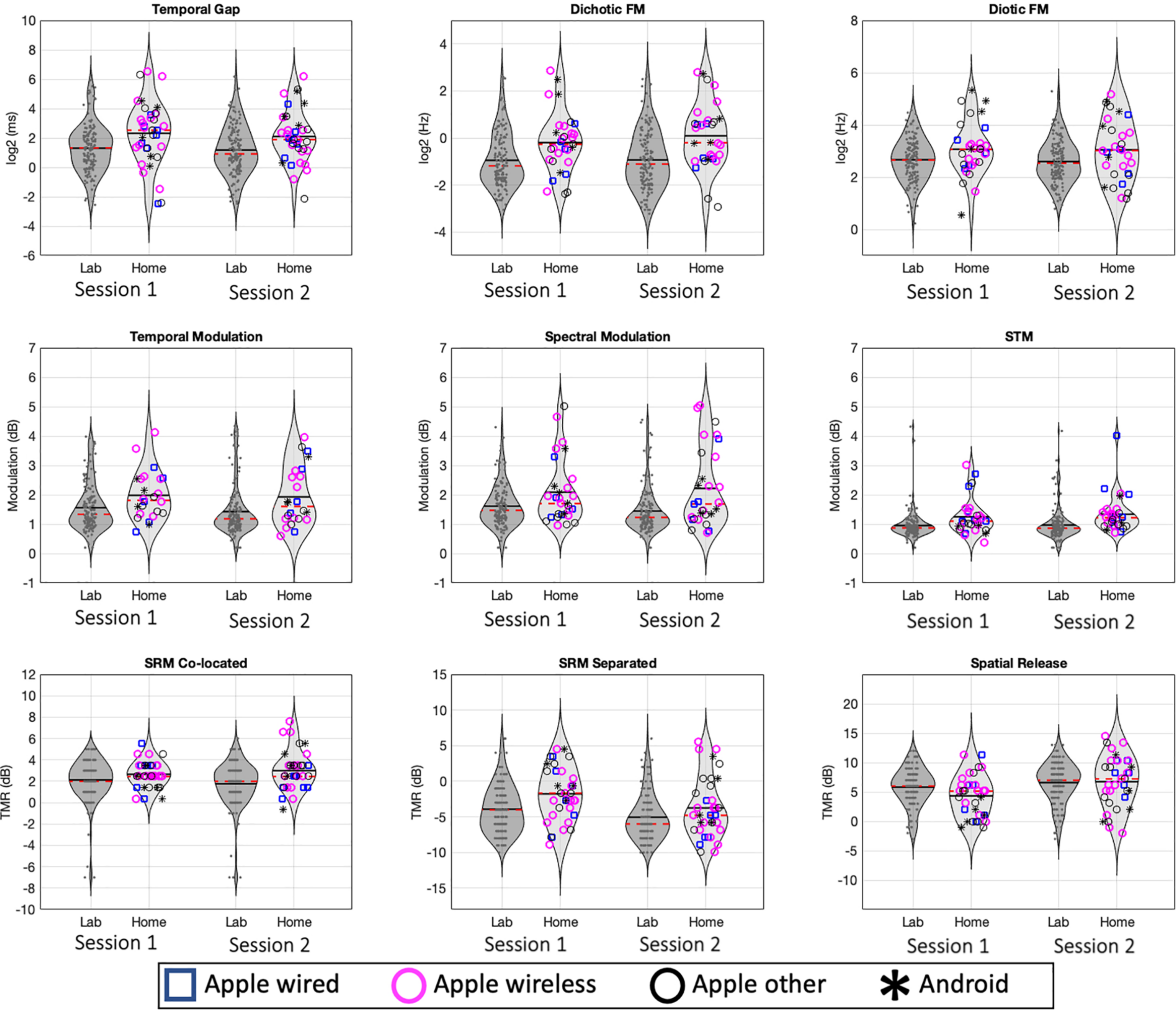
(Color online) Density functions and scatter plots of individual participant datapoints for experiment 1 (lighter distributions) and Larrea-Mancera *et al.* (2020) (darker). Dashed lines inside each density function represent medians, and solid lines represent means, of each session's distribution (note, in most cases, solid and dashed lines overlap).

Figure 1 presents the distributions of thresholds obtained in the two sessions of experiment 1 (“home”; lighter distributions) compared with the normative data sets of Larrea-Mancera *et al.* (2020) (“lab”; darker distributions). Symbols indicate different hardware used by participants (combinations of iOS and Android devices and wired or wireless headphones). A mixed-effects analysis of variance (ANOVA) with the between-subject factor of experiment (HOME vs LAB) and within-subject factor session (ONE vs TWO) was computed for each of the nine assessments and the spatial release metric. Relevant statistics, including *p* values, are presented in Table I. Small but statistically significant main effects of experiment (*p *≤* *0.01) were observed for each assessment, with the exception of the spatial release metric.

**TABLE I.** Data from experiment 1 (@home) compared to the normative dataset (lab). Columns show mean data and SD for each session and difference between sessions. The replication column shows results for the mixed-model ANOVAs (main effect of experiment) of each assessment. Effect sizes are shown for partial *η*^2^ and Cohen's *D.*

Assessment	Mean (SD) session 1	Mean (SD) session 2	Mean difference	Replication *f (p)*	*df*	*η_p_* ^2^	Cohen's *D*
Gap (lab)	2.46 (3.15)	2.26 (3.18)	2.22 ms	11.74 (<0.01)*	183	0.06	0.5
@home	4.5 (4.59)	4.66 (3.97)					
DichoticFM (lab)	0.51 (2.23)	0.52 (2.37)	0.37 Hz	13.96 (<0.01)*	179	0.07	0.55
@home	0.82 (2.48)	0.98 (2.68)					
DioticFM (lab)	6.35 (1.75)	6.05 (1.77)	2 Hz	7.7 (<0.01)*	177	0.04	0.41
@home	8.16 (2.005)	8.24 (2.25)					
TM (lab)	1.55 (0.81)	1.42 (0.85)	0.43 M(dB)	7.69 (<0.01)*	170	0.04	0.42
@home	1.92 (0.82)	1.92 (1.005)					
SM (lab)	1.61 (.72)	1.44 (.78)	0.61 M(dB)	18.22 (<0.01)*	170	0.09	0.65
@home	2.06 (1.07)	2.21 (1.34)					
STM (lab)	0.95 (0.46)	0.96 (0.55)	0.32 M(dB)	13.96 (<0.01)*	165	0.07	0.56
@home	1.24 (0.61)	1.31 (0.64)					
Colocated (lab)	2.12 (1.96)	1.76 (2.08)	1.1 TMR(dB)	13.97 (<0.01)*	181	0.07	0.55
@home	2.89 (1.58)	3.2 (1.96)					
Separated (lab)	−3.91 (3.32)	−5.04 (3.2)	1.86 TMR(dB)	11.9 (<0.01)*	183	0.06	0.51
@home	1.81 (3.68)	−3.4 (4.37)					
Spatial release (lab)	5.8 (3.24)	6.58 (3.37)	0.77 dB	2.17 (0.28)	176	0.01	0.22
@home	4.43 (3.38)	6.41 (4.44)					

The differences observed between experiment 1 and the normative dataset in most measures are approximately half a SD in magnitude (Cohen's *D* from 0.38 to 0.65). Effect sizes, expressed in original units, correspond to changes in threshold for the remote testing of 2.2 ms for the gap task, 0.3 Hz for dichotic FM, 2 Hz for diotic FM, from 0.32 to 0.61 modulation nominal dB for STM, and between –1.49 and 1.86 TMR (nominal dB) differences in the targets in competition assessments (see Table I). At first glance, these results would appear to suggest that remote administration in home environments using personal equipment yields worse performance than the laboratory conditions of the original study (however, this conclusion will be revisited after the data of the subsequent experiments are considered).

### B. Are remotely administered auditory thresholds repeatable and reliable?

In this section, we address two key questions relevant to the feasibility of remote auditory testing: First, to what extent are measures repeatable across sessions? Second, are differences between subjects reliable across sessions? High reliability, as was observed with the normative dataset, is essential for development of measures that can be used to compare across individuals.

**FIG. 2.**
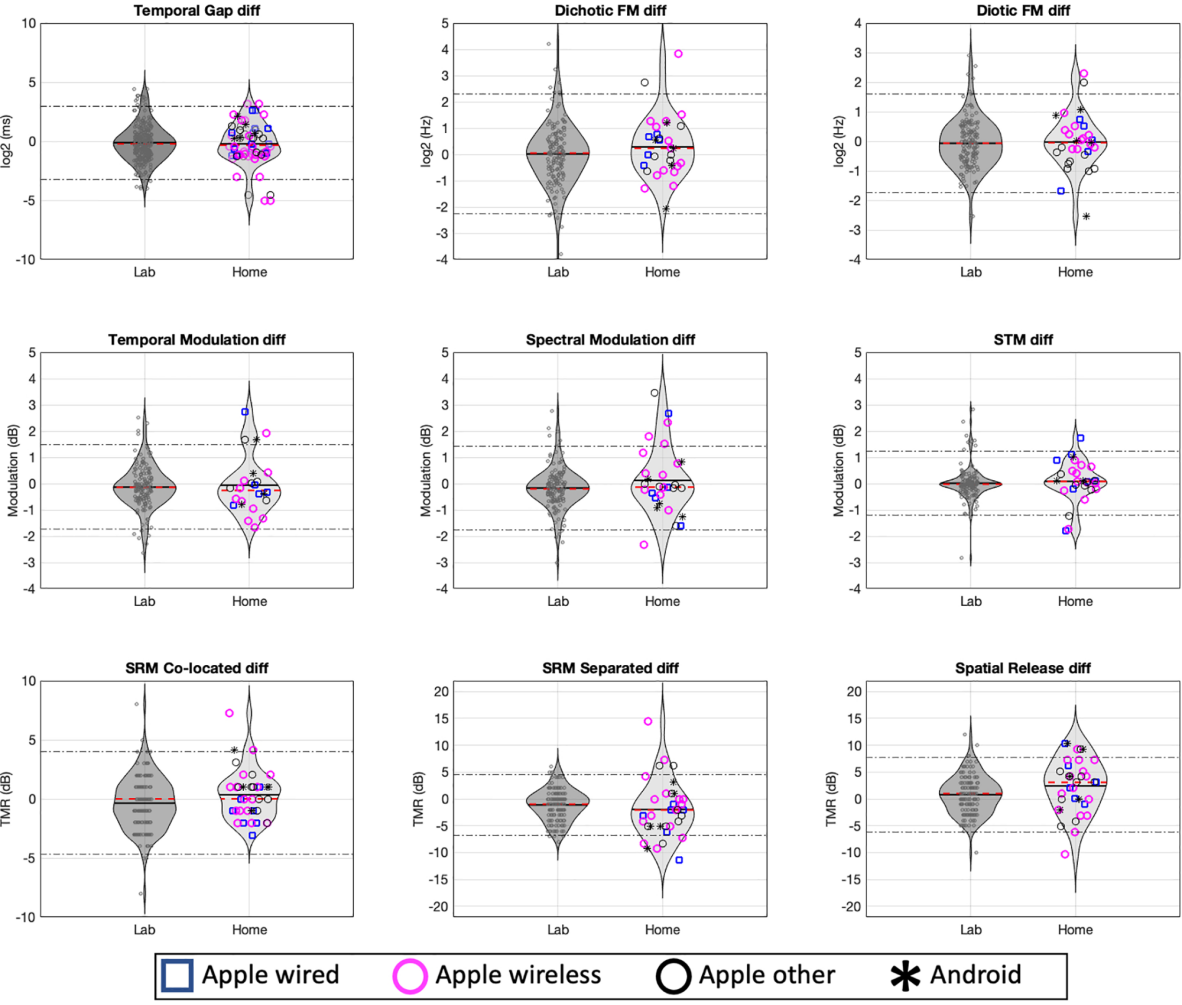
(Color online) Density functions for differences between sessions (session 2 – session 1) from experiment 1 (lighter distributions) and for normative dataset (darker) and this study. The dashed line inside each distribution represents the median, and the solid line represents the mean of each study. Dark dotted lines represent the 95% limits of agreement extracted from the normative dataset, which establish expectations for most of the differences between repeated measures to occur.

To address repeatability, we first examined the interaction term (experiment × session) of the ANOVA (see Table II) to test whether the between session differences (small but significant) were different at home as compared to in the laboratory. None of the interactions between experiment and session was statistically significant, suggesting that differences between sessions were not reliably greater at home than in the laboratory. To quantify the size of these effects, difference scores were calculated by subtracting session 1 thresholds from session 2 thresholds. The SD of the difference scores multiplied by the critical value of 1.96 yields a coefficient of reliability (COR) according to Bland and Altman (1999). This COR was used to establish a range around the mean difference between sessions (denoted bias) and to denote the limits of agreement. These limits represent the parametric region where 95% of the differences between sessions are expected to occur. The limits of agreement (LoA) of the normative dataset (Larrea-Mancera *et al.*, 2020) are shown as dotted lines in Fig. 2. The LoA are used to quantify reliability and can be used to compare home to laboratory testing in this experiment and to compare both home and lab testing to the normative data set. Again, different symbols are used to indicate the classes of participant-owned hardware used. Of note, the interaction term shown in Table II is statistically equivalent to subjecting the difference scores to a one-way ANOVA with experiment as factor (LAB vs HOME). Thus, a lack of interaction is indicative of a lack of significant experiment factor on the difference between sessions. Taken with the LoA shown in Fig. 2, this suggests that repeatability with personally owned equipment was similar to repeatability found in the lab-based normative study with calibrated equipment.

**TABLE II.** Differences between mean thresholds in session 1 and session 2 for experiment 1 (home) and the normative study (lab; Larrea-Mancera *et al.*, 2020). Except for spatial release, negative values indicate an improvement from session 1 to 2. The repeatability column shows results for the mixed-model ANOVA interaction term (experiment * session) of each assessment. For all assessments, the *F* values are not statistically significant (*p* > 0.05). Partial *η*^2^ are estimates of the variance captured by the interaction, and Cohen's *D* expresses the size of the difference in units of SD.

Assessment	*Δ Session lab*	*Δ Session home*	Repeatability *F* (*p* value)	*df*	*η_p_* ^2^	Cohen's *D*
Gap	−0.2 ms	0.16 ms	0.32 (0.57)	183	0.002	0.08
DichoticFM	0.01 Hz	0.16 Hz	1.2 (0.27)	179	0.007	0.16
DioticFM	−0.3 Hz	0.08 Hz	0.25 (0.61)	177	0.001	0.07
TM	−0.13 M (dB)	0.007 M (dB)	0.57 (0.45)	170	0.003	0.11
SM	−0.17 M (dB)	0.15 M (dB)	2.99 (0.08)	170	0.01	0.26
STM	0.01 M (dB)	0.07 M (dB)	0.21 (0.64)	165	0.001	0.06
Colocated	−0.36 TMR (dB)	0.31 TMR (dB)	2.96 (0.08)	181	0.01	0.25
Separated	−1.13 TMR (dB)	−1.59 TMR (dB)	0.52 (0.47)	183	0.003	0.1
Spatial release	0.78 dB	1.98 dB	2.73 (0.2)	176	0.005	0.24

### C. Experiment 1 summary and equipment effects

**FIG. 3.**
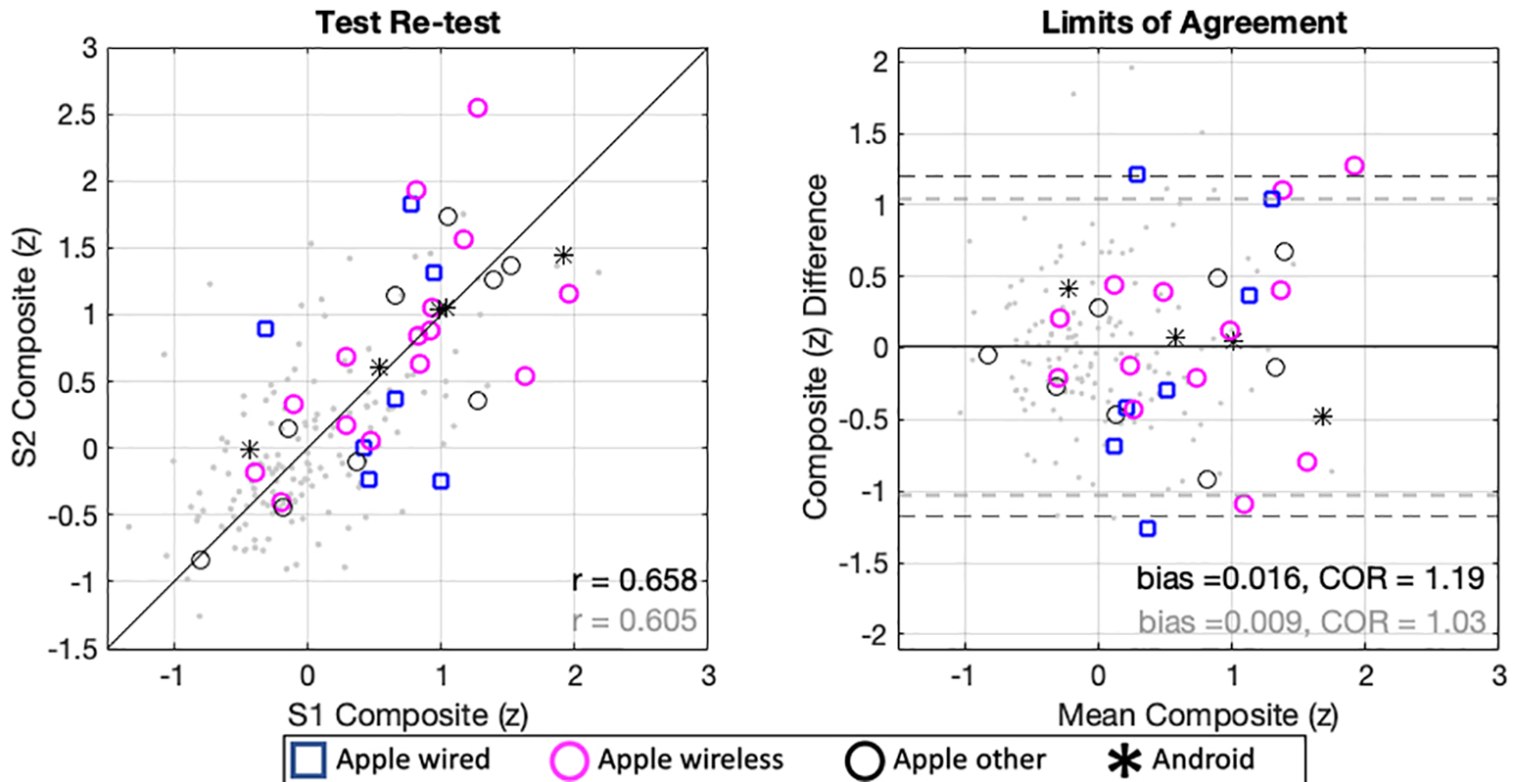
(Color online) Left panel shows correlation across session for composite scores of both experiments (symbols indicate different device and headphone combinations). Normative dataset composite scores are depicted in light gray. *r* values (gray for normative dataset) are significant (*p <* 0.001). Right panel shows the mean of sessions plotted against their differences. The mean of differences (bias) and the limits of agreement are displayed as a solid and dotted lines, respectively. The COR computed from the SD of differences is also displayed. Again, gray data correspond to the normative dataset.

To further interpret the findings across all remote measures and to test the possible effects of the different devices and headphones used, a composite score was calculated for each subject on each session as the average of z-scores in all nine assessments, using the means and SDs from the normative dataset (Larrea-Mancera *et al.*, 2020). Figure 3 (left panel) depicts the strength and direction of association between scores obtained in each session for experiment 1 (HOME, multiple symbols) and the normative data set (LAB, light symbols). Figure 3 (right panel) also shows the LoA as well as the bias and COR. These figures suggest that average performance was slightly worse than in the normative dataset, but reliability across sessions was very similar. To quantify this, we compared Pearson normalized correlation values obtained in each study (LAB *r = *0.65; HOME *r = *0.67) with a Fisher z-test and failed to observe any statistically significantly difference (*z =* –0.19, *p = *0.42).

Figure 3 (both panels) shows effects of the device and headphones used. While there was considerable variability in this regard, the preponderance of participants used iOS devices and Apple insert headphones. As a result, there was insufficient statistical power to conduct meaningful significance testing contrasting devices or headphones even for the broad categories we used: (1) iOS device, Apple wired headphones (*n* = 7); (2) iOS device, Apple wireless headphones (*n* = 15); (3) iOS device, other headphones used (*n* = 9); and (4) Android device, other headphones used (*n* = 5). Data related to these equipment categories are shown using different markers for composite scores (Fig. 3) and across all tests in terms of threshold (Fig. 1) and reliability (Fig. 2). To evaluate possible differences across equipment categories with a test of significance, a mixed-effects ANOVA was conducted, with the within-subject factor session (ONE vs TWO) and the between-subject factor device+headphone (four levels: iOS+wired, iOS+wireless, iOS+other, Android+other). No significant main effects were found for either device+headpone [*F*_(3,32)_ = 0.31, *p* = 0.81] or session [*F*_(1,32)_= 0.002, *p* = 0.96]; nor was there a statistically significant interaction [*F*_(3,32)_ = 0.08, *p* = 0.81].

## IV. EXPERIMENTS 2 AND 3

### A. Procedure of experiments 2 and 3

To better understand the differences found in the mean performances on tests of auditory processing, we conducted two additional within-subject experiments comparing participant-owned equipment in one session and calibrated equipment in another (order counterbalanced between subjects). Calibration ensures that the stimuli as measured by the calibration system have the desired overall presentation level and the nominal level at different audio frequencies and that the output at the two transducers is balanced. Ultimately, momentary transducer placement on the subject determines whether these parameters hold during testing. Thirty-six new participants completed testing remotely (experiment 2), and another 58 completed testing in the laboratory (experiment 3; same rooms used as in the normative dataset: Larrea-Mancera *et al.*, 2020). Procedures in experiment 3 differed from experiments 1 and 2 only in that testing was in the lab, and thus research assistants monitored sessions in person rather than using the video-chat tool. This design allowed further investigation of the impact participant-owned devices might have in the estimated thresholds, which was an unknown factor in experiment 1 and a potential cause of the threshold differences from the normative data set. Furthermore, participants were asked to use a questionnaire to report the level of auditory and visual distractors in the environment and their ability to maintain focus in each session.

## V. RESULTS OF EXPERIMENTS 2 AND 3

Section V A addresses whether variability can be explained in terms of the equipment used or the location where testing was conducted. Section V B explores reliability of measures in the additional experiments. Section V C addresses self-report data about distractors and focus and its capacity to account for measure variability. Finally, Sec. V D shows the distribution of outliers across settings. A summary of the most important experimental design details can be found in Table III.

**TABLE III.** Summary details of all three experiments.

Experiment	*N*	Equipment used	Test setting
Larrea-Mancera *et al.* (2020)	150	Calibrated lab devices	Laboratory
Experiment 1	40	Participant-owned uncalibrated devices	Remote
Experiment 2	36	Calibrated/uncalibrated devices	Remote
Experiment 3	58	Calibrated/uncalibrated devices	Laboratory

### A. Can the differences found be explained by either device or location?

**FIG. 4.**
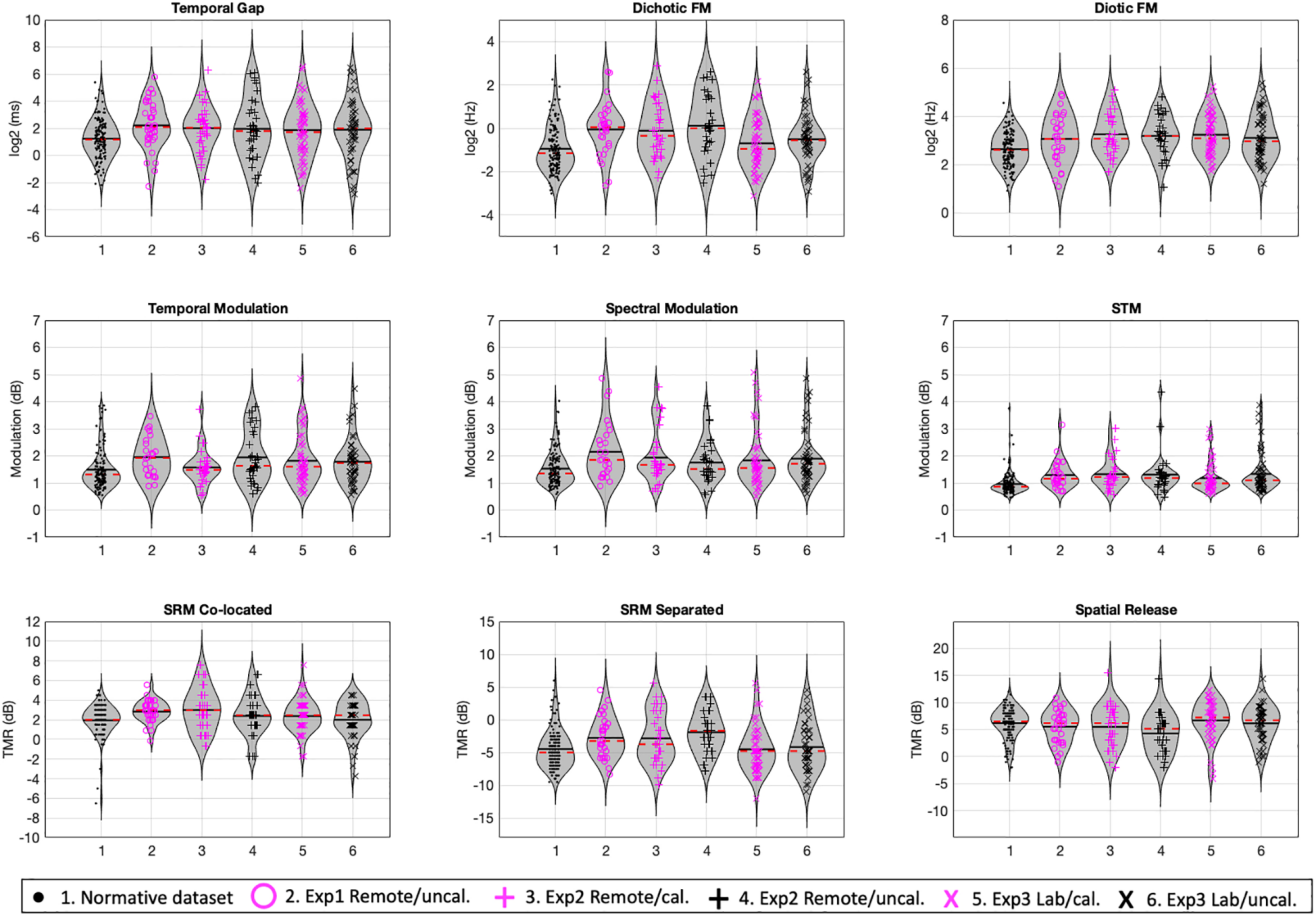
(Color online) Density functions and spread of datapoints for the normative dataset (average of sessions), experiment 1 (average of sessions), and experiments 2 and 3 divided by system used (calibrated lab-based or uncalibrated participant-owned). The dashed line inside each density function represents the median, and the solid line represents the mean of each distribution.

Figure 4 shows the distribution of estimated thresholds of the normative dataset (average between sessions; Larrea-Mancera *et al.*, 2020) followed by the average between sessions of experiments 1, 2, and 3 separated based on equipment used (personally owned or laboratory-based). To formally test for differences between these measures that may be explained by device, location, or their interaction, we conducted a mixed-model multivariate analysis of variance (MANOVA) with within-subject factor equipment (CALIBRATED vs PERSONALLY OWNED) and between-subject factor location (REMOTE vs LAB) for all eight measures (excluding the spatial release metric) of experiments 2 and 3. We obtained non-significant contributions of each factor (equipment: *Pillai trace *=* *0.097, *p *=* *0.102; location: *Pillai trace *=* *0.092, *p *=* *0.127) and a non-significant interaction (*Pillai trace *=* *0.047, *p *=* *0.127) for equipment and location. The MANOVA analysis thus provides no evidence that either equipment or location can account for the differences found in the contrast between experiment 1 and the normative data set.

### B. Was the reliability of measures consistent with previous experiments?

**FIG. 5.**
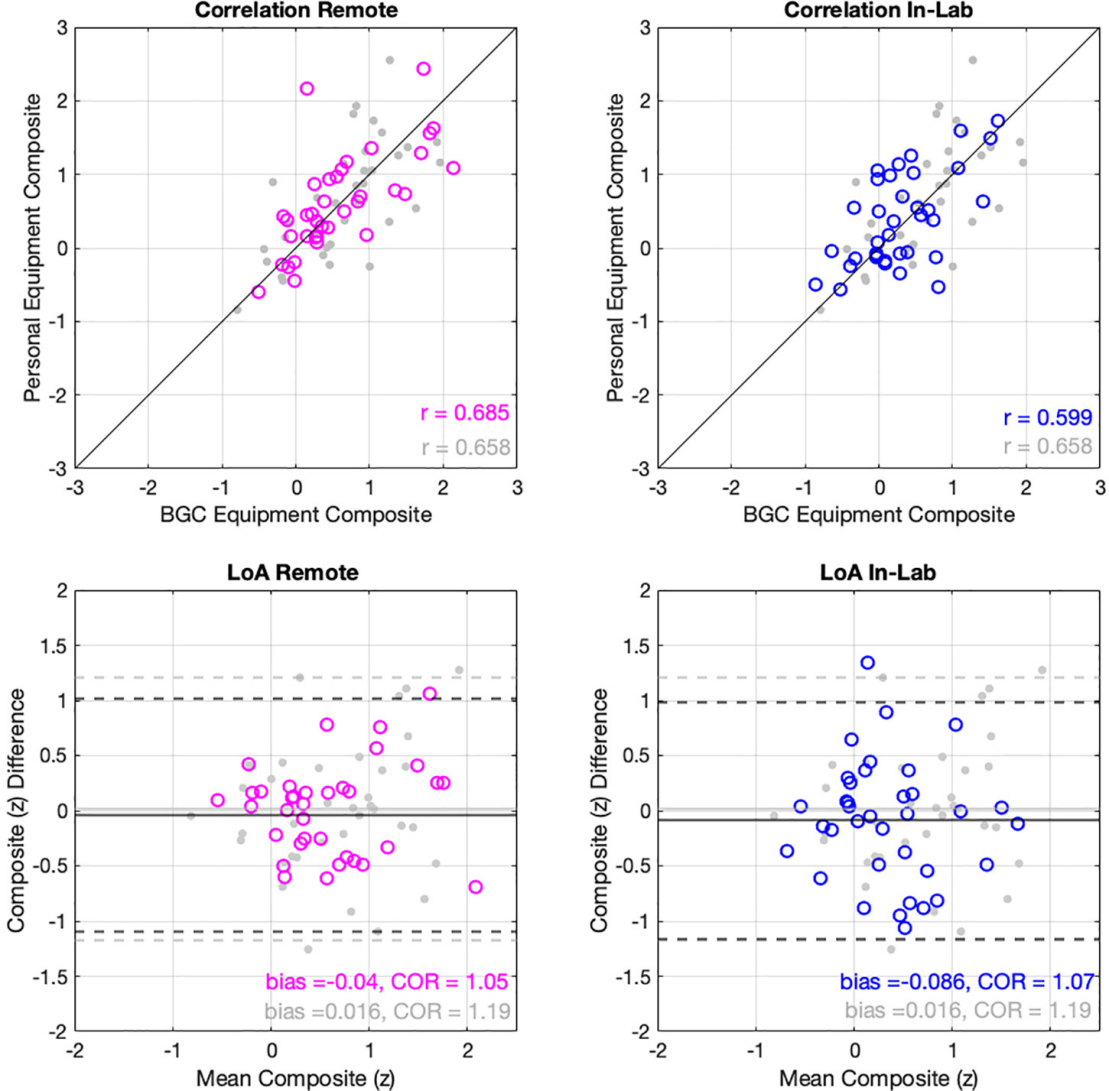
(Color online) Correlation between sessions using different equipment (top panels) for experiment 2 (conducted remotely; left panels), and experiment 3 (conducted in-lab; right panels). Experiment 1 data are shown in gray for comparison. Limits of agreement and the bias (mean difference) are shown as solid and dotted lines, respectively (bottom panels).

Figure 5 shows correlations and limits of agreement of the standardized composite scores from experiments 2 and 3. Reliability was again similar to that found in the normative dataset, with no differences in correlation between sessions 1 and 2 between either experiment 2 (*z *=* *0, *p *=* *0.5) or experiment 3 (*z *=* *0.71, *p *=* *0.23) and the normative dataset. Further, limits of agreement in experiments 1 and 2 are close to a SD, and both samples show very small bias to either session. Of note, composite scores shown in Fig. 5, which were standardized to the normative dataset, are not centered at zero, reflecting a similar difference to that found in experiment 1 (displayed in gray behind experiment 2 and 3 distributions).

### C. Does self-reported distraction and focus explain variance?

To better understand differences in performance observed between laboratory and remote testing, in experiments 2 and 3, participants were asked to report visual, auditory, or verbal distractors as well as their level of focus using a Likert scale (0–5). To estimate contributions of these self-report measures, we computed correlations against the composite score obtained from the average of the standardized scores of each test of each participant (see supplementary Fig. S7, top row).^1^ None of the correlations were significant for any of the equipment or location conditions. To test whether differences in these self-report scores explained differences in performance across sessions, we analyzed whether a change in the self-reported distractors and focus correlated with a change in the composite score. This correlation of change scores (self-report vs composite; see supplementary Fig. S7, bottom row)^1^ also failed to show significant effects. As such, we were unable to find reliable evidence that environmental distractors or individual focus explained variance in the estimated thresholds in either the controlled or uncontrolled environments.

### D. Outlier analysis

Following the observation that there were more outlying cases in experiment 1 than in the normative dataset, analyses were conducted to explore whether outlying performance occurred more frequently at home or in the lab. Figures. S2 and S3 in the supplementary materials^1^ illustrate the outliers found in each of experiments 2 (remote) and 3 (in-lab). The number of rejected outlier cases for each task was as follows: none for the gap, for the TM (remote: 6; lab: 9), for SM (remote: 4; lab: 9), for STM (remote: 8; lab: 11), for dichotic FM (remote: 6; lab: 5), for diotic FM (remote: 3; lab: 8), none for the SRM colocated, and two for the SRM separated (remote: 1; lab: 1). Most of the outlying performance datapoints are restricted to a single session without a clear bias toward a type of equipment or setting. This can be corroborated in Figs. S1–S3 of the supplementary materials.^1^

## VI. DISCUSSION

The results of this study indicate that reliable data on a range of measures of auditory processing ability can be collected remotely using participant-owned equipment, at least for young adults with normal hearing sensitivity. While data for each of the tasks were somewhat worse in replication studies compared to the original normative dataset (Larrea-Mancera *et al.*, 2020), test-retest reliability was similar. To better understand the differences found, experiments 2 and 3 examined potential impacts of uncalibrated devices and location (lab vs remote). Neither factor nor their interaction made a significant difference across experiment 2—conducted remotely—nor experiment 3—conducted in the lab. Further, we failed to find evidence that self-reported environmental distractors or participant focus explained significant variance. Thus, overall, there were similar thresholds found for the three new datasets collected (see Fig. 5), suggesting that differences from the normative dataset may be a cohort effect rather than simply a remote testing disadvantage.

We note that the data in this study were collected during the COVID-19 pandemic, whereas the normative dataset was collected prior to the pandemic. It has been suggested that the COVID-19 pandemic can be conceptualized as a multi-dimensional stressor that impacts several spheres of people's lives (Wirkner *et al.*, 2021) including cognitive measures (Fiorenzato *et al.*, 2021; Favieri *et al.*, 2021). For example, in adolescents, the pandemic period has been associated with increased stress and self-reports of cognitive difficulties (Attwood and Jarrold, 2022). Moreover, the relationship between social isolation and cognitive impairment has been previously reported (Cacioppo and Hawkley, 2009) and has even been linked to an increased risk for early mortality, suggesting generalized impacts on the human condition (Holt-Lunstad *et al.*, 2015). Ingram *et al.* (2021) studied the effect of COVID-19-induced social isolation in several cognitive tasks and found that performance declined with social isolation and improved as individuals had opportunities to socialize. These observations were replicated in a binational study with different styles of social lockdown (Ingram *et al.*, 2021). Considering the evidence above, it is possible that collecting data during the pandemic might have resulted in a general reduction in performance among this undergraduate sample. However, we note that it is also possible that the original normative dataset was non-representative, and further data collection will be required to establish whether the original dataset or these new datasets better represent long-term norms.

It is notable that reliability was similar across all experiments, even when using participant-owned equipment. The exploration of device variability impacts in experiment 1, albeit underpowered, yielded no indication of device effects. These results replicate and extend the findings of Larrea-Mancera *et al.* (2020) to show that the PART produces reliable data when used with a wider range of devices and headphone types than were previously tested. This finding was also confirmed in experiments 2 and 3, where in neither remote nor laboratory settings were we able to find uncalibrated system effects. Thus, these data indicate that precise and accurate calibration might not be essential for the suprathreshold tests investigated here, although calibration cannot be completely ruled out as a factor that contributes to the remaining unexplained variance in our data.

The robustness of threshold estimation across different types of equipment in all of the assessment results is bolstered by three additional factors. First, some of the chosen measures were previously known to be robust to substantial variations in overall level [SM: Isarangura *et al.* (2019); TM: Viemeister (1979)], while level effects are unexplored for the other methods. Second, analyses of differences in the current data and normative data for tasks in which stimuli differed in bandwidth (broadband, such as TM, SM, STM, and SRM, vs narrowband, such as FM) or channels (dichotic, such as FM and SRM, vs diotic, such as gap, TM, SM, and STM) indicate that variations in within- or across-system channels did not lead to systematic differences in results. Third, in Larrea-Mancera *et al.* (2020), differences in output levels greater than 10 dB between the two headphone types tested did not affect thresholds or variability. These are exciting results as they suggest it is adequate to harness participant-owned technologies for psychophysical testing toward gathering large datasets of diverse auditory abilities.

We also considered self-reported measures of visual, auditory, and verbal distraction as well as participant focus as additional factors that might account for the differences between the normative dataset and experiment 1. Even though all our testing sessions were monitored via videocalls to ensure participants were performing the task as instructed (e.g., wearing headphones), providing some additional confirmation that there were not major distractions during the experiment, we were concerned that a difference in distractors and focus would still manifest across test settings. The self-reported measures, however, were not correlated with performance. We further analyzed if changes in the self-reported scores correlated with changes in repeated measures and failed to identify a significant association. Environmental factors are difficult to quantify, and future studies would benefit from ambient sound monitoring that could more rigorously estimate the extent to which trial-by-trial variability in the local auditory environment might impact psychometric testing. For example, Kopun *et al.* (2022) monitored environmental noise in a remote testing setting by instructing participants to evaluate the average environmental noise level after each block of testing and only continued testing if the environmental noise level did not exceed 50 dBA SPL. Troubleshooting instructions were given in case the 50-dBA SPL cutoff was exceeded to ensure more acceptable environmental noise levels for testing. However, while ambient noise could explain some differences in estimated thresholds, previously, no significant effect on performance was found when cafeteria noise was played through a loudspeaker at 70 dBA SPL during assessments carried out in PART (Larrea-Mancera *et al.*, 2020). Of note, the headphones used in that study have 30 dB passive attenuation.

We also explored the potential effect of outliers. In experiment 1, we noted a greater proportion of outlying scores—more than 3 SDs worse than the mean of the normative data—suggesting that the less controlled at-home environment may occasionally lead to spurious test results. These outlying values do not seem to be directly related to the general device types used as performance within the expected range was found both on iOS and Android and both wired and wireless headphones. However, the increased presence of outliers was probably due to the shift in mean performance in contrast to the normative dataset whose parameters were used to carry out the outlier rejection. Indeed, if the new experiment parameters are used for rejection, the number of cases per test does not exceed 2. This observation was replicated with experiment 2 and experiment 3, both of which had a similar number of outliers with respect to each other, but more than the normative dataset. Importantly, we note that other than in a few cases, most outliers are inconsistent between sessions, and a third measurement could be used to clarify whether the outlier datapoint reflects poor auditory performance or whether it is more related to some sort of attentional lapse or other failures to respond to the test properly. Consistent outlying performance can be easily identified, and participants with difficulties can be brought into more controlled conditions for further testing in the absence of potential extraneous variables.

Regarding clinical screening and monitoring, it is important to consider the relationship between false-positives and false-negatives in remotely administered auditory testing used for clinical purposes. We suggest that it is unlikely that participants would perform in the normal range at home but then show impairment in the clinic. Under this framework, remote testing can complement the necessary but less accessible clinical procedures. For example, poor performance on a remotely administered test can serve to motivate an in-person visit to gain more reliable data. Further, when at-home data match in-clinic data, regular remote monitoring would be an efficient and cost-saving option compared to clinical monitoring. Next steps will involve validation of these remote testing procedures for people with hearing difficulties to test the extent to which remote testing provides clinically relevant information.

The results of this study demonstrate the potential of conducting remote auditory testing using people's own devices in their home environments. Valid and reliable remote testing could increase access to clinical assessment, addressing both clinical and research needs. Advantages of remote testing are manifold, where lower costs and greater accessibility can help in generating larger, more representative, datasets of hearing function in the general population; increasing sample diversity; and including systematically underserved populations. The devices already distributed among the general public seem to have reached a quality level necessary to achieve valid assessment of auditory function. The use of this accessible technology can accelerate data collection and facilitate the inclusion of a more diverse set of participants in clinical research studies. However, we note that this might not be the case in countries or regions where people have less access to current technologies for their personal use. To address this, as well as to meet the needs of research studies that might require a stricter experimental control in this regard, one could easily distribute calibrated devices at lower costs than in-person clinical assessment (as was done in experiment 2). We acknowledge, however, that this study represents only a first step in establishing the utility of remotely testing auditory function, as similar validation studies will also be required for individuals with hearing impairment. Further, it will be necessary to address the relative utility of introducing additional controls, such as ambient sound monitoring and more precise assessment of the at-home environment, that could increase the reliability of auditory measures within home environments. Still, we suggest that the data presented in this paper provide compelling evidence for the feasibility of remote auditory testing, which, if more broadly utilized, could transform auditory research and clinical practice alike.

## Footnotes

^1^
See supplementary material at https://www.scitation.org/doi/suppl/10.1121/10.0013221 for additional analysis on the reported datasets.
